# Relative abundance and distribution of fisheries influence risk of seabird bycatch

**DOI:** 10.1038/srep37373

**Published:** 2016-11-23

**Authors:** Andrea Soriano-Redondo, Verónica Cortés, José Manuel Reyes-González, Santi Guallar, Juan Bécares, Beneharo Rodríguez, José Manuel Arcos, Jacob González-Solís

**Affiliations:** 1 Institut de Recerca de la Biodiversitat (IRBio), Departament de Biologia Evolutiva, Ecologia i Ciències Ambientals, Universitat de Barcelona, Av. Diagonal 643, 08028 Barcelona, Spain; 2 Centre for Ecology and Conservation, College of Life and Environmental Sciences, University of Exeter, Cornwall Campus TR10 9EZ, UK; 3 SEO/BirdLife - Marine Programme, C/Murcia 2-8, local 13. 08026 Barcelona, Spain; 4 Canary Islands’ Ornithology and Natural History Group (GOHNIC), La Malecita s/n, 38480 Buenavista del Norte, Tenerife, Canary Islands, Spain

## Abstract

Fisheries provide an abundant and predictable food source for many pelagic seabirds through discards, but also pose a major threat to them through bycatch, threatening their populations worldwide. The reform of the European Common Fisheries Policy (CFP), which intends to ban discards through the landing obligation of all catches, may force seabirds to seek alternative food sources, such as baited hooks from longlines, increasing bycatch rates. To test this hypothesis we performed a combined analysis of seabird-fishery interactions using as a model Scopoli’s shearwaters *Calonectris diomedea* in the Mediterranean. Tracking data showed that the probability of shearwaters attending longliners increased exponentially with a decreasing density of trawlers. On-board observations and mortality events corroborated this result: the probability of birds attending longliners increased 4% per each trawler leaving the longliner proximity and bird mortality increased tenfold when trawlers were not operating. Therefore, the implementation of the landing obligation in EU waters will likely cause a substantial increase in bycatch rates in longliners, at least in the short-term, due to birds switching from trawlers to longliners. Thus the implementation of the landing obligation must be carefully monitored and counterbalanced with an urgent implementation of bycatch mitigation measures in the longline fleet.

Effects of fishing on marine megafauna are widespread and diverse, mainly due to overfishing, production of discards and bycatch^1^,^2^,^3^. Bycatch, the incidental capture of non-target species, is of particular concern for long-lived species with low reproductive rates and delayed sexual maturity, such as seabirds^1^. Baited hooks offer the opportunity for an easy meal, yet these entail a very high risk of birds being hooked and subsequently drowned. A recent global review estimated seabird bycatch by longlines in 160,000–320,000 birds per year^4^. In fact, for some species, the current rates of bycatch are unsustainable for their long-term viability^5^,^6^,^7^,^8^.

On the other hand, fishery discards may also have profound impacts on the breeding biology, distribution and population dynamics of seabirds, by making available demersal and benthonic species otherwise naturally inaccessible^9^,^10^,^11^. Worldwide discards are estimated to be 8% of the total catch (i.e. around 7,000,000 tonnes discarded annually^2^). Ultimately, discards seem to be responsible for the increases in population sizes of several scavenging species over the last decades, such as large gulls^9^,^12^.

Seabird-fishery interactions are of particular concern in the Mediterranean^13^; an enclosed and low-productive sea with a high degree of endemism^14^. Four seabird species are endemic to the basin and commonly caught by fishing gear, particularly in longlines: the Audouin’s gull *Larus audouinii,* and the Scopoli’s *Calonectris diomedea*, Yelkouan *Puffinus yelkouan* and Balearic shearwaters *Puffinus mauretanicus*^15^,^16^. Among these, the Yelkouan and Balearic shearwaters are globally threatened (Vulnerable and Critically Endangered, respectively^17^). According to conservative estimates, at least 5,000 birds could be killed annually in the region^18^. In particular, the bycatch of Scopoli’s shearwaters represents over 50% of all birds caught in longlines in some areas of the Western Mediterranean, which would imply that 4–6% of the local population breeding in the Balearic Islands is killed every year^4^,^19^,^20^. For these species, bycatch by other gears appears to be far less relevant in the region^15^,^16^.

In the Mediterranean Sea, discards are estimated to be 18% of the catch (i.e. around 230,000 tonnes discarded annually), with trawlers being responsible for 15 to 65% of the discards^18^. Discard availability modifies the diet, foraging strategies and distribution of seabirds^21^,^22^,^23^,^24^, with some species obtaining up to 75% of their energy needs from this resource^25^,^13^.

To further complicate matters, interactions among different fisheries may lead to unexpected indirect effects on seabirds. Some recent studies in the Mediterranean provided evidence that the attendance of Scopoli’s shearwaters to trawlers and longliners may depend on the relative activity schedules of these fleets^26^,^27^,^28^. When trawlers do not operate, shearwaters may seek alternatives to discards, such as baits used by longline fisheries, with a consequent increase in the risk of being hooked^27^,^28^. This possibility needs to be fully explored, since changes in fishery schedules or discard availability can occur at any time, and a proper management may minimize their negative impacts on seabird mortality. Indeed, the reform of the Common Fisheries Policy (CFP, http://ec.europa.eu/fisheries/cfp/) by the European Union (EU), among other measures, is implementing the elimination of discards through the so called *landing obligation*, with the aim of reducing the impact of fisheries on marine ecosystems. Therefore, there will be a gradual reduction in discards, from now to 2019, that could severely affect the Mediterranean seabird community, including the threatened shearwater species^13^. Thus, there is an urgent need to improve our understanding about the undesirable interactions among different fishery activities on seabird mortality.

To cast light on this problem, we studied the interaction between Scopoli’s shearwater and fishing boats, and how different fishery schedules and vessel distribution patterns affect bycatch on the western Mediterranean Sea. Specifically, we used three different approaches: (1) individual GPS trajectories of Scopoli’s shearwaters and Vessel Monitoring System (VMS) trajectories of fishing boats to study spatiotemporal dynamics, and establish whether vessel densities determine shearwaters choice between longliners and trawlers; (2) seabird counts on-board longliners to determine the main drivers influencing seabird attendance during longline settings, focusing particularly on the potential influence of trawler activity in the surrounding area; and (3) 13 complete years of bycatch data from one longline vessel to understand whether the rate of bycatch increases on days when trawlers do not operate.

## Results

### Spatiotemporal interactions

**Figure 1:**
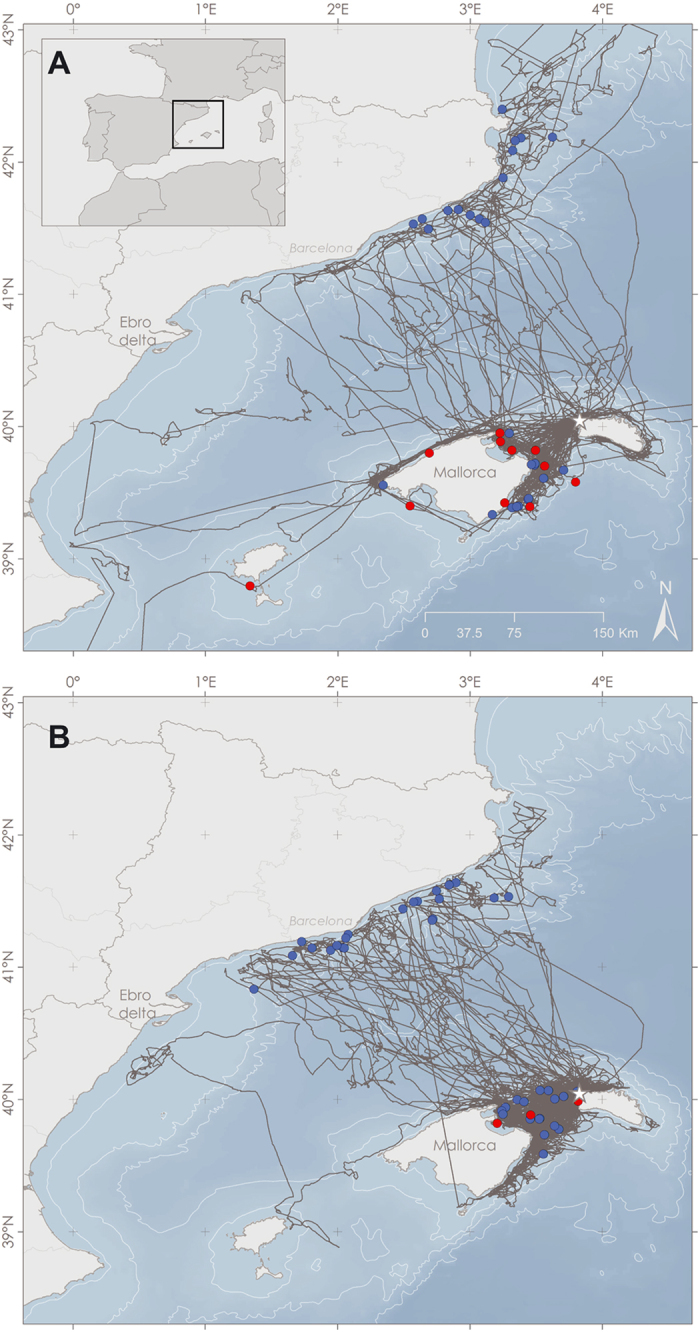
Shearwater GPS tracks (in grey) and concurrent interactions between shearwaters and fishing vessels (dots) inferred from the Vessel Monitoring System (VMS) in 2010 (**A**) and 2012 (**B**). Red dots correspond to interaction with longliners and blue dots to interactions with trawlers. Maps were generated with ArcGis version 10.3 (URL: https://www.arcgis.com).

**Figure 2:**
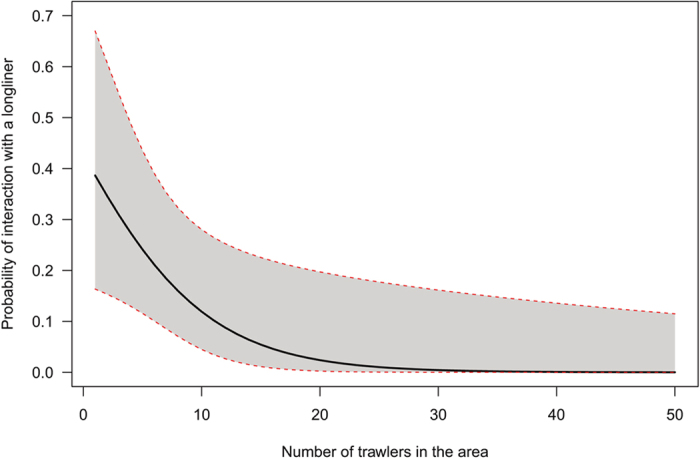
Probability of interaction between shearwaters and longliners as a function of the number of trawlers in the area. Black line represents the predicted values from the model and the grey area represents the 95% confidence intervals.

Overall, we tracked 65 shearwaters in two different years, 2010 and 2012, with 4 birds being tracked both years. We obtained trajectories from 90 GPS deployments, 30 trajectories corresponding to 38 foraging trips in 2010 and 60 trajectories corresponding to 145 foraging trips in 2012 (Fig. 1). Birds mainly foraged in the Catalan shelf and the Menorca channel, areas used by both trawlers and longliners (Fig. 1). We obtained 267 interaction events, where a bird followed a vessel, 246 interactions occurred with trawlers and 21 with pelagic longliners. From those, only 86 events corresponded to events in which at least one longliner and one trawler were fishing simultaneously in the same area (Fig. 1). From those interactions, 72 were with trawlers and 14 with longliners. Interactions with longliners mainly happened in the Menorca channel, close to the breeding areas, while interactions with trawlers happened both in the Catalan shelf and in the Menorca channel. We found no direct effect of the number of longliners in the area on the probability of interaction with either a trawler or a longliner. However, the probability of interacting with a longliner increased as the number of trawlers decreased (P = 0.029), from nearly 0% when 20 or more trawlers were in the area, to 40% probability of interaction when only 1 trawler was present (Fig. 2).

### On-board censuses

We found that the number of attacks to the bait and the number of birds following a vessel was correlated (r_s_ = 0.38, P = 0.004). Since sample size for seabird attendance was greater than for bait attacks, we used seabird attendance as a proxy for bycatch risk on subsequent analyses, allowing us to study the ultimate factors that influence it. Analysis of relative importance showed that setting time was the main variable to account for shearwater presence and abundance behind longliners: shearwaters were more likely to occur in greater numbers at twilight (Tables 1 and 2). Bait type and longline type also had an important effect on bird attendance, birds being more likely to interact with longliners when they used mixed baits (fish and cephalopods, instead of only one of both) and when they targeted pelagic species (instead of demersal species) (Tables 1 and 2). The distance to the nearest breeding colony had a relatively high influence on shearwater abundance; the further away from colony areas the less likely it was to detect birds attending longliners (Tables 1 and 2). The number of hooks in each setting had a very low effect on the presence and abundance of birds attending longliners (Tables 1 and 2). The breeding stage of the birds (pre-laying, incubation or chick-rearing) and the meteorological conditions did not affect bird attendance to longliners (Tables 1 and 2). The probability of birds attending longliners increased 4% per each trawler leaving the longliner surroundings, but bird abundance was not affected by the number of trawlers (Tables 1 and 2).

### Bycatch data

From 2003 to 2015, we collected all birds hooked in a longline boat from Vilanova i la Geltrú after arrival to the port. During this period, 67 Scopoli’s shearwaters became entangled in the fishing gear and died. We found that the number of birds caught differed significantly among the days of the week; it was higher on Sundays and Mondays than on the remaining days (χ^2^ = 17.63, p < 0.001). In fact, 51% (34 individuals) of the birds were hooked on Sundays, when trawlers do not operate, and 28% (19 individuals) on Mondays, when longline vessels start operating before trawlers after the weekend rest. From Tuesday to Friday, only 14 birds were hooked across the 13 years covered. In addition, we found that the probability of a bycatch event (when one or several birds were hooked) followed the same pattern: events were more likely to occur on Sundays and on Mondays than on the rest of the week days (12 bycatch events occurred on a Sunday, 5 on a Monday and 6 from Tuesday to Friday; χ^2^ = 4.9, p = 0.03).

## Discussion

The concurrent analysis of GPS data from seabirds and vessels showed the importance of the spatiotemporal distribution of operating trawlers in determining the probability of seabirds interacting with longliners: the higher the density of trawlers, the less likely birds interacted with a longliner. On-board censuses of seabird attendance to longliners corroborated this result, showing that attendance, which can be taken as a proxy of bycatch risk, increased when trawlers were not operating in the longliner proximity. Finally, dead birds collected by fishermen showed that seabird catches were significantly and substantially greater on days when trawlers did not operate. Hence, results from these three different approaches point towards the same direction: seabird bycatch in longliners significantly increases when trawlers operate in low densities or do not operate.

In this regard, the reform of the CFP, which intends to substantially reduce or even eliminate fishery discards, might dramatically increase seabird bycatch risk, at least in the short term, by forcing seabirds to intensify their foraging efforts, including the search of an “easy meal”, i.e. switching from trawler discards to longline baits. Our results show that, at present, trawlers are acting as a buffer of the seabird interactions with longliners, such that the probability of shearwaters interacting with a longliner decreases from 40% when only one trawler is present in the area to almost 0% when >20 trawlers are present (Fig. 2). The same pattern applies to the on-board observations and bycatch rates. For each trawler leaving the surroundings of a longliner, the probability of seabirds following the longliner increases by 4%; and the rate of bycatch experiences a tenfold increase when trawlers do not operate. The reduction of discards is therefore likely to result in a substantial increase in bycatch rates of Scopoli’s shearwaters, to a level that could be completely unsustainable for some western Mediterranean populations^29^. Moreover, our results can reasonably be extrapolated to other seabirds species targeting trawler discards in the Mediterranean which are also known to be caught in longlines, such as the Audouin’s gull and the Yelkouan and Balearic shearwaters^23^. The latter is of particular concern given its sensitive conservation status, since bycatch appears to account for almost half the adult mortality estimated for the species^8^.

Given the concern of bycatch for many Mediterranean seabirds, including the four endemic species, it is urgent to take into account this multi-fisheries interaction when designing fishing regulations, in order to minimise its potentially detrimental effect. As a first approach, our results suggest that precluding longline vessels (both demersal and pelagic) to set their lines when trawlers are not operating might substantially contribute to this aim. However, given that (trawling) discards will anyway be reduced in the short run, our results also call for an immediate enforcement of effective mitigation measures in longliners to reduce seabird bycatch. Since our results also point out that the strongest influence on seabird attendance to longliners was the time of setting, operational measures regulating the setting timing should contribute to minimise the problems. Indeed, seabirds were more prone to interact with longliners during sunset and sunrise. These results have also been observed in other studies which showed that many diurnal seabirds have activity peaks at dawn and dusk^19^,^27^,^30^. Since most seabirds affected by longliners in the Mediterranean are basically diurnal, a promising mitigation measure to be applied in this region would be night setting, as previously suggested in other studies^19^,^30^,^31^,^32^,^33^,^34^. This measure could be easily implemented at low economic costs, and compliance could be monitored and enforced to some extent through the control of fishing schedules of longliners by harbour authorities, as it is currently done for other types of fisheries. However, fishermen could be reluctant because it would require a rearrangement of their schedules and it could also limit the number of setting operations, particularly during the relatively short summer nights. Therefore, further work is needed to assess the efficacy and viability of this measure as well as of other mitigation measures that have proven successful in other regions. Among them, the use of tori-lines, the increase of sinking rates of the line through configuration changes, or a combination of the above, might also contribute to minimise seabird bycatch in Mediterranean longliners^31^,^34^,^35^,^36^,^37^.

In conclusion, our study highlights the importance of combining various sources of information to achieve robust and complementary results on the complex effects of fishing activities on seabird bycatch. In particular, three different approaches indicated that the risk of a seabird to be captured in longlines increases dramatically when trawlers are not present in the area where longliners operate. That is, when trawlers stop providing discards seabirds may switch from trawlers to longliners, and therefore the landing obligation being implemented by the CFP must be carefully monitored and counterbalanced with the urgent implementation of mitigation measures. In a more general sense, our results point out that to determine the best management practices of the different fishing fleets, we need to study unexpected impacts rising from the interactions among different types of fisheries. Therefore, impacts of changes on the discard availability must be carefully evaluated and monitored across the different fleets to avoid catastrophic effects on seabird populations as well as on other components of the marine ecosystem.

## Methods

### Spatiotemporal interactions

To establish the spatiotemporal interaction between tracked birds and fisheries, we obtained data from two main sources: GPS devices for tracking shearwaters and the VMS for tracking vessels. GPS-tracking of Scopoli’s shearwaters was conducted in Cala Morell (Menorca, Balearic Islands, Spain; 40°3′N, 3°52′E), in two different years. In 2010, birds were tracked during the incubation period, from the 18^th^ June to the 8^th^ July; and in 2012 during the chick rearing period, from the 25^th^ July to the 20^th^ September. This area holds the largest Scopoli’s shearwater colony of the Balearic Islands, tentatively estimated in 1,000–6,000 pairs^38^,^39^. Scopoli’s shearwaters were captured at night by hand or using looped poles on the nest, when they flew back to the colony to feed their offspring or for incubation shifts. We used GPS loggers (Perthold Engineering LLC, weighing 20 g^40^) sealed to be waterproof and programmed to record bird position each 2.5 or 5 minutes. Loggers were attached to the back of the birds using Tesa© tape^41^. At deployment, birds were ringed, sexed (through biometric measures) and weighed. In an attempt to minimise adverse effects on the birds, total mass of the device did not exceed 3% of the birds body mass^42^. At recovery, GPS devices were detached and birds were weighed. On average, we found a 25 g decrease on bird weight after tag retrieval (Paired t-test, t = 4.4138, df = 83, p < 0.001), that we do not expect to have relevant effects on the foraging behaviour of the birds^43^. In 2010, we deployed 30 GPS tags in 25 individuals, whereas in 2012, 79 GPS loggers were deployed on 56 individuals. In order to minimise the possible impact of tagging birds on their breeding success, only one adult bird per nest was tagged. Birds carried loggers from 3 to 17 days before retrieval, recording from 1 to 9 foraging trips. These protocols were approved by *Servei de Protecció d’Espècies*, from the Balearic Islands Government. The methods were carried out in accordance with the relevant guidelines and bird handling and tagging protocols.

The Vessel Monitoring System (VMS) is a satellite-based monitoring system, implemented by the European Union, that provides data on the location, course and speed of fishing vessels over 12 m long^44^. In the Spanish Mediterranean, 90% of trawlers and 60% of pelagic longliners use this localization system, although only a few demersal longliners and artisanal (polyvalent) vessels use it^45^. The default frequency of VMS locations is one fix every two hours. Consequently, the spatiotemporal combination of VMS and GPS data was obscured by the uncertainty about the position of each vessel in the two hour gap between two consecutive locations. Taking into account this limitation, to cover the potential interactions between birds and vessels throughout the entire vessel trip, we identified all bird locations within a ±1 h interval and within a 5 km buffer from a vessel location (as maximum speeds of vessels are around 5 km/h). Next, we applied a second filter selecting bird trajectories where the bird bearing diverged in less than ±30ᵒ from the vessel bearing (estimated from consecutive locations). This bird bearing was considered the mean bearing for all locations inside each buffer of spatiotemporal coincidence. We chose ±30ᵒ because after some trials this angle emerged as the most biologically meaningful figure. For each bird-vessel interaction location, we assessed the number of vessels within a ±1 h interval and a 30 km radius. We chose 30 km as it has been shown that some procellariforms can detect food resources up to 30 km away^46^,^47^. We focused our research in two types of fisheries, trawling and pelagic longlining, since shearwaters tend to associate with them in search of food. Demersal longliners, including artisanal (polyvalent) vessels were excluded from this analysis, despite also attracting (and catching) seabirds, since most of them are too small to carry the VMS system (see above). Moreover, we only selected the interaction events where at least one trawler and one longliner were present in the area to control for the fisheries different regime and ensure that when birds interacted with a trawler they had also the option to interact with a longliner and vice versa.

We used a generalized lineal mixed model that included year as a random effect, number of trawlers and number of longliners as fixed effects and a binomial response, either the bird interacted with a longliner or it interacted with a trawler. We calculated the AIC values of all candidate models and selected the model with the lowest AIC value as the best model for explaining bird interaction with fisheries.

### On-board censuses

At-sea surveys were carried out during 3 consecutive years (2011–2013) covering the main fishing grounds of longline fisheries in the Catalan shelf and Balearic Islands, during the period where the species was present in the Mediterranean (March–October). The counts were conducted during 102 longline settings from 20 small-scale vessels operating in the NW Mediterranean (16 demersal and 4 pelagic longliners). Here we only considered the maximum number of Scopoli’s shearwaters following the vessels at the end of each 10-min counts in each setting operation, as well as the number of attacks to the bait and the number of birds incidentally captured. The number of birds hooked (26 birds in nine events) was too low to perform reliable statistical analyses. Fishing habits, detailed description of fishing gear used and meteorological data were noted in each fishing trip (Table 2). The number of trawlers in a 6 km radius around the longliner was also recorded in each 10-min survey.

Bycatch events are relatively rare and patchy, thus being difficult to monitor through low-effort observer programmes. We evaluated seabird attendance to longliners as a proxy of seabird attacks, which are more closely related to seabird bycatch and therefore mortality. Generalized lineal mixed models (GLMMs) were used to identify the main factors influencing Scopoli’s shearwater interaction with longliners. Abundance data is characterized by having a high proportion of zero values and a skewed distribution of non-zero positive values caused by large counts of individuals (flocking behaviour)^48^. Hurdle models are a suitable method for modelling this type of distributions, which is characterized by treating the data in two parts: (1) presence/absence of the species (Zero part) and (2) the abundance when the species is present (Count part)^48^,^49^,^50^,^51^. We analysed the relationship between the number and presence of Scopoli’s shearwaters and temporal, spatial and operational variables (Table 2). Trawler presence in the surrounding area was also considered to assess their influence on seabird attraction to longliners. Longliner identity was used as a random effect. We used “glmmadmb” function from the “glmmADMB” R package (R version 3.1.2). Zero hurdle part was modelled with the assumption of a binomial error structure (logit link function), while in the Count part a truncated version of the Negative Binomial distribution was considered (log link function). We checked collinearity between predictors and removed redundant ones. Then, we used the variance inflation factor (VIF) to verify the independence of each variable on the estimate of the regression coefficients of the model^52^.

Relative importance analysis was carried out with the model-averaging approach. This approach is useful when there is a large uncertainty about a set of models^53^. In this way, we obtained model-averaged parameter estimates that were directly comparable to each other^53^. We estimated the parameters from the set of all models for which the sum of Akaike weights reached >0.95.

### Bycatch records

From 2003 to 2015 fishermen from a single demersal longline vessel fishing off the Catalan coast recorded and handed over Scopoli’s shearwater carcasses accidentally caught in their longline. To establish temporal bycatch patterns, we analysed whether the number of birds hooked and the number of capture events differed among the days of the week by using a Pearson’s Chi-squared Test for Count Data with Bonferroni correction.

## Additional Information

**How to cite this article**: Soriano-Redondo, A. *et al*. Relative abundance and distribution of fisheries influence risk of seabird bycatch. *Sci. Rep.* **6**, 37373; doi: 10.1038/srep37373 (2016).

**Publisher's note:** Springer Nature remains neutral with regard to jurisdictional claims in published maps and institutional affiliations.

## Figures and Tables

**Table 1:** Coefficient, standard errors and relative importance (RI) of each variable for shearwaters attendance to longliners.

Variable	Value	Std. Error	RI
*Presence - Zero part* (Intercept)	−0.84	1.01	
Bait: Fish	1.75	1.11	0.93
Bait: Mixed	2.73	1.38	
LT: Pelagic	1.96	1.31	0.59
Time: Daytime	−1.39	0.79	0.90
Trawlers	−0.18	0.24	0.53
SDC	−0.27	0.45	042
Period: Incubation	0.11	0.47	0.35
Period: Prelaying	−0.41	0.74	
Wind: Windy	−0.07	0.34	0.24
SHS	0.02	0.16	0.24
*Abundance – Count part* (Intercept)	2.48	0.47	
LT: Pelagic	0.15	0.40	0.31
Time: Daytime	−0.53	0.46	0.76
Trawlers	−0.01	0.09	0.22
SDC	−0.15	0.19	0.55
Period: Incubation	0.01	0.07	0.05
Period: Prelaying	0.01	0.11	
Wind: Windy	−0.03	0.16	0.20
SHS	−0.08	0.14	0.39

See Table 2 for detailed explanation of the variables.

**Table 2:** Explanatory variables used in the analyses of Scopoli’s shearwaters interaction with longliners.

Variable	Abbreviation	Type	Description
Breeding period	Period	Categorical	Pre-laying (March – 1st week June), incubation (until mid-July) or chick-rearing (until October)
Distance to colony	SDC	Continuous	Distance from the nearest colony (scaled km)
Wind	Wind	Categorical	Windy or still
Bait composition	Bait	Categorical	Fish, cephalopods or mixed (fish + cephalopods)
Setting time	Time	Categorical	1 h ± of the twilight or rest of the daytime
Longline type	LT	Categorical	Demersal or pelagic
Hooks setting	SHS	Continuous	Number of hooks setting (scaled)
Number of trawlers	Trawlers	Continuous	Number of trawler within 6 km from the longliner
Longliner ID		Random	Boat identifier
